# Management of the Sequelae of a Sport-Related Traumatic Dental Injury Using Ultrasound Examination in the Diagnosis and Follow-Up

**DOI:** 10.3390/dj9030027

**Published:** 2021-03-02

**Authors:** Davide Musu, Giulia Bardini, Francesca Ideo, Silvia Mezzena, Elisabetta Cotti

**Affiliations:** Department of Conservative Dentistry and Endodontics, University of Cagliari, 09124 Cagliari, Italy; supergiu.gb@gmail.com (G.B.); ideofrancesca@gmail.com (F.I.); silvia@studio-mezzena.it (S.M.); cottiendo@gmail.com (E.C.)

**Keywords:** apical periodontitis, ultrasound examination, sport, traumatic dental injury

## Abstract

About a quarter of all oral pathologies involving the oral cavity and dental apparatus are traumatic injuries, and a substantial number of these cases are the result of sports injuries affecting adolescents and young adults. Here, we report the case of a 25-year-old healthy female referred to the department of Endodontics for the evaluation and management of teeth 1.2 and 1.1 because of a chronic apical abscess in an area involved in a sport-related dental trauma in the past. A multi-modular diagnostic assessment, comprising conventional periapical radiographs, CBCT imaging, ultrasound, and histopathologic examination, led to a final diagnosis of an apical granulomatous lesion connected to both teeth, and an associated sinus tract. During the follow-up period of three years, the patient was reviewed twice a year and showed progressive healing of the bone and absence of the sinus tract. The present report shows the challenges of diagnosing complications arising from past dental trauma. Furthermore, it is the first documented traumatic case where ultrasound examination was fruitfully used. Emphasis should be put on introducing diagnostic ultrasound for the management of both apical periodontitis and the related sinus tract.

## 1. Introduction

Traumatic injuries constitute almost a quarter of all pathologic conditions that involve the oral cavity and teeth, and a substantial number of these cases result from sports injuries affecting adolescents and young adults [1]. Indeed, within this age range, there is a peak of athletes who start practicing agonistic sports, most of which involve body contact and are connected with a higher risk of trauma to the teeth and oral cavity [2]. Some of the consequences of these injuries appear years after the time of the trauma, and may become challenging to diagnose and treat. In a study on sport-related dental trauma conducted on a sample of athletes, it was demonstrated that 5 years after the traumatic event there was a 25% incidence of pulp necrosis, especially in luxation cases where the periodontal ligament is often involved [3]. A further complication that may arise following the loss of pulp vitality is the development of apical periodontitis (AP) in the involved teeth [4]. AP is highly prevalent in the general population, and is defined as an inflammatory disease involving the periapical tissues, caused by the host immune response to microorganisms in the infected root canal system [5]. When AP develops, the prognosis for healing, following a correct endodontic treatment, is reduced by 15–20% [6] when compared to a regular root canal treatment, thus complicating the delicate situation of traumatized teeth. Furthermore, AP mainly manifests as a periapical cyst or granuloma, and it has been hypothesized that cysts are less likely to heal with respect to granulomas [7]. It is, therefore, important that an adequate diagnostic rationale is available for the clinician to assess the periapical status of traumatized teeth. Ultrasound examination, also called ultrasonography or echography, is an advanced imaging technique based on the propagation and reflection of ultrasound waves into the tissues of the body [8]. This technique was introduced into endodontics for the first time in the early 2000s, when it was demonstrated that it was possible to obtain echographic images of apical periodontitis, and to gain information related to the content of the lesions [9]. Subsequently, ultrasonography has been enhanced with the color-power Doppler (CPD), which has proved useful to differentiate between granulomatous and cystic apical lesions using histopathology as the reference standard [10,11], and to monitor the healing of AP after both surgical and non-surgical treatment [12,13]. The aim of the present report is to discuss a case where the sequelae of a sport-related traumatic injury were diagnosed and followed-up with a multi-modular imaging approach. The rationale for this is that novel diagnostic technologies, such as ultrasound examination, can be useful in the management of the endodontic consequences of traumatic injuries by visualizing the features of the periapical area.

## 2. Case Report

**Figure 1:**
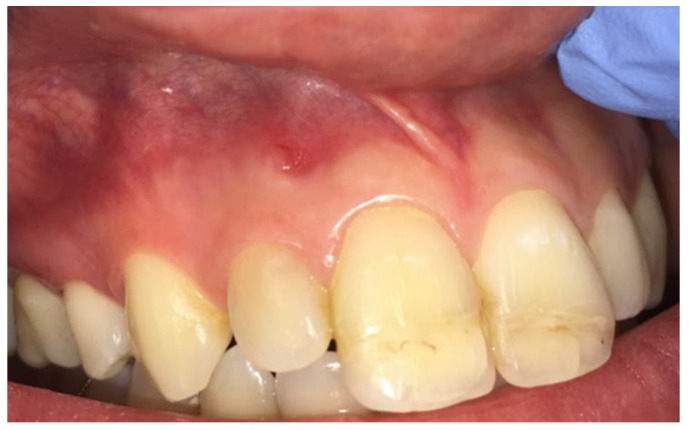
Clinical examination showing coronal integrity of tooth 1.2, discoloration of teeth 1.1 and 2.1, and the stoma of a sinus tract located apically between teeth 1.1 and 1.2.

**Figure 2:**
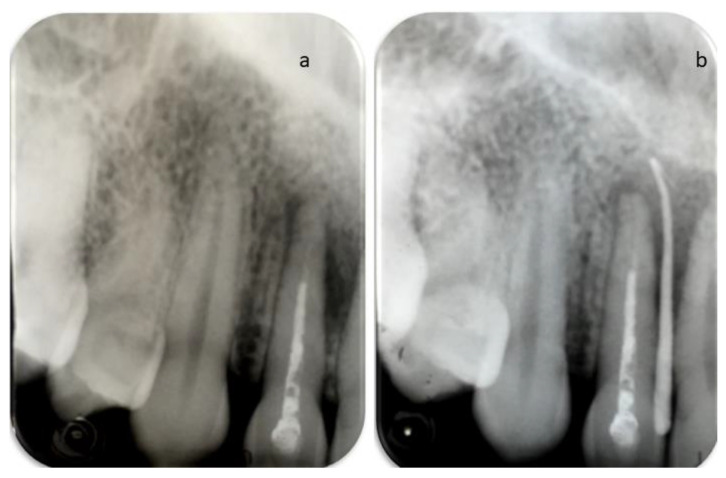
(**a**) Periapical radiograph depicting the presence of a previous root canal treatment on tooth 1.2 and a periapical radiolucency. (**b**) Periapical radiograph performed with insertion of a gutta-percha cone within the stoma to trace the sinus tract.

A 25-year-old Caucasian female patient was referred to the Department of Endodontics of the University of Cagliari, with the chief complaint of periodical drainage of purulent material from the opening of a sinus tract located in the buccal mucosa, apical to teeth 1.2 and 1.1, in the fall of year 2017. The sinus tract had appeared a few months earlier. The patient’s medical history was positive for chronic bronchial asthma, and non-contributory for other diseases or medications. The patient reported a sport-related dental trauma due to a body contact with another player, which occurred six years earlier (2011) when she was an agonist volleyball player. As a consequence of that trauma, she reported the enamel-dentinal fracture of teeth 1.1 and 2.1 and the concussion of tooth 1.2, according to the IADT guidelines [14]. In 2014 (3 years after the trauma) she developed an acute apical abscess on tooth 1.2, and a root canal treatment was then performed. Starting in 2017, the patient manifested a periodical discharge of pus in that same area, that was managed with antibiotic therapy and self-performed drainage for a while. The day of our visit, besides the active pus discharge, the patient referred to tenderness of tooth 1.2 when chewing. Upon clinical examination, the presence of two discolored restorations on teeth 1.1 and 2.1 were noted, as well as the stoma of a sinus tract in the alveolar mucosa apical to teeth 1.1 and 1.2 (Figure 1). The clinical exams performed on tooth 1.2 showed tenderness to palpation, percussion, and the bite test, and no periodontal probing, while tooth 1.1 showed an uncertain response to the thermal testing (cold test) and no other signs or symptoms. A periapical radiograph, taken with the paralleling technique, depicted the presence of a root canal treatment, looking not adequate, as well as a periapical radiolucency on tooth 1.2 (Figure 2), and no apparent lesions or widened periodontal ligament on tooth 1.1. A subsequent radiograph with a gutta-percha cone introduced in the opening of the tract traced the route of the drainage, showing the periapical lesion on tooth 1.2. as the source for this condition (Figure 2).

**Figure 3:**
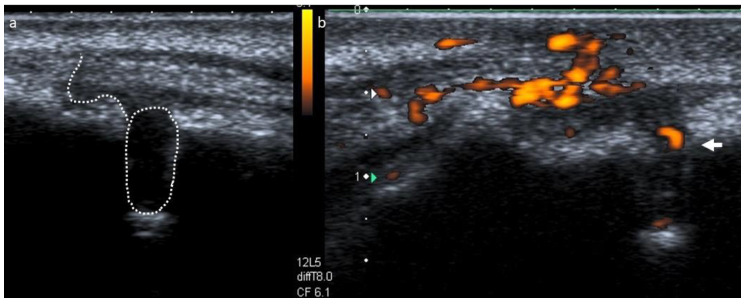
(**a**) B-mode ultrasound examination showing a sinus tract disrupting the vestibular bone, presenting as a dishomogeneous, hypoechoic pathway lined by echogenic and reinforced contours. (**b**) Power Doppler applied to the same area, showing an echogenic, solid lesion with poorly defined hyperechoic bone boundaries and internal blood vessels.

To assess the content and vascular supply of the lesion, a real-time ultrasound examination with the addition of CPD was performed (Toshiba Aplio XG, Toshiba Medical Systems, Crawley, UK, and a regular size, linear, high definition, multi frequency ultrasound probe at 8–12 MHz). The exam displayed an echogenic, solid lesion with a poorly defined hyperechoic bone outline and internal blood vessels, suggestive of an apical granuloma [10] (Figure 3). This examination also portrayed an ecographic representation of the sinus tract, which interrupted the cortical plate and exhibited a dishomogeneous, hypoechoic pathway surrounded by echogenic and reinforced boundaries (Figure 3). Our diagnosis was of a pulpless tooth with a chronic apical abscess. The treatment plan included orthograde root canal retreatment of tooth 1.2, followed by root-end surgery if an adequate quality of retreatment could not be achieved [15], alongside the continuous monitoring of tooth 1.1.

**Figure 4:**
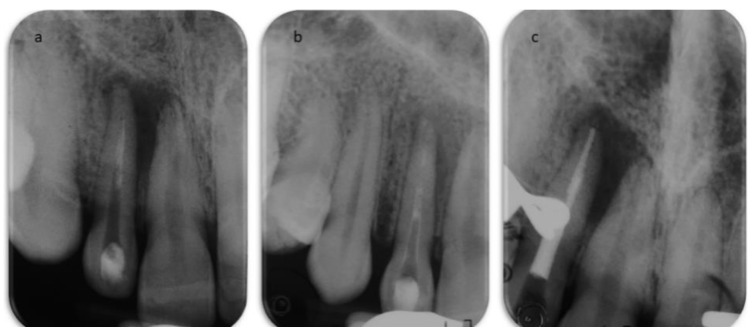
Root canal treatment. (**a**) Periapical radiograph after the first access to the root canal system. (**b**) Radiograph showing the calcium hydroxide temporary dressing within the canal after the first visit. (**c**) Periapical radiograph after obturation of the root canal system.

**Figure 5:**
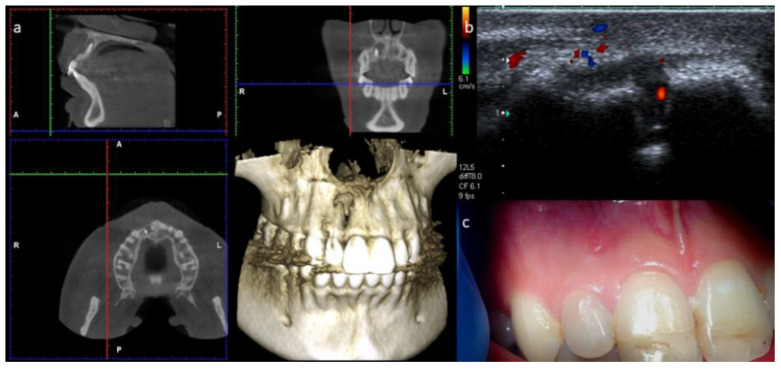
Three-month follow-up. (**a**) CBCT depicting the extent of the lesion and the extensive bone loss involving the periapical area of teeth 1.2 and 1.1. (**b**) Color Doppler examination showing the persistence of both the sinus tract and the inflammatory vascular signal within the lesion. (**c**) Clinical picture showing the persistence of the sinus tract.

**Figure 6:**
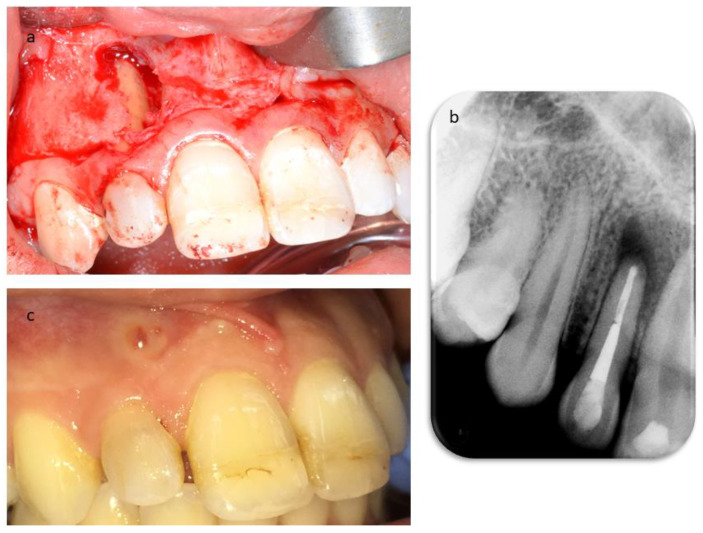
(**a**) Intra-operative picture of the surgical endodontic treatment. (**b**) Post-operative periapical radiograph. (**c**) Clinical examination of the patient at one month after surgery showing the persistence of the stoma.

**Figure 7:**
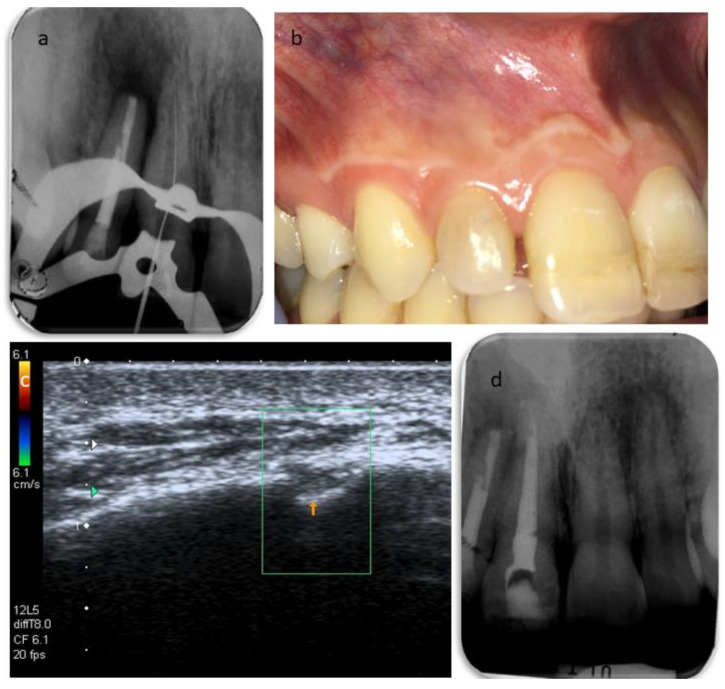
(**a**) Root canal treatment of tooth 1.1. (**b**) Clinical examination at one week after the root canal treatment, showing the disappearance of the stoma. (**c**) Ultrasound examination with the addition of the color Doppler showing the disappearance of the sinus tract and the vascular signal within the periapical lesion. (**d**) Post-operative periapical radiograph.

**Figure 8:**
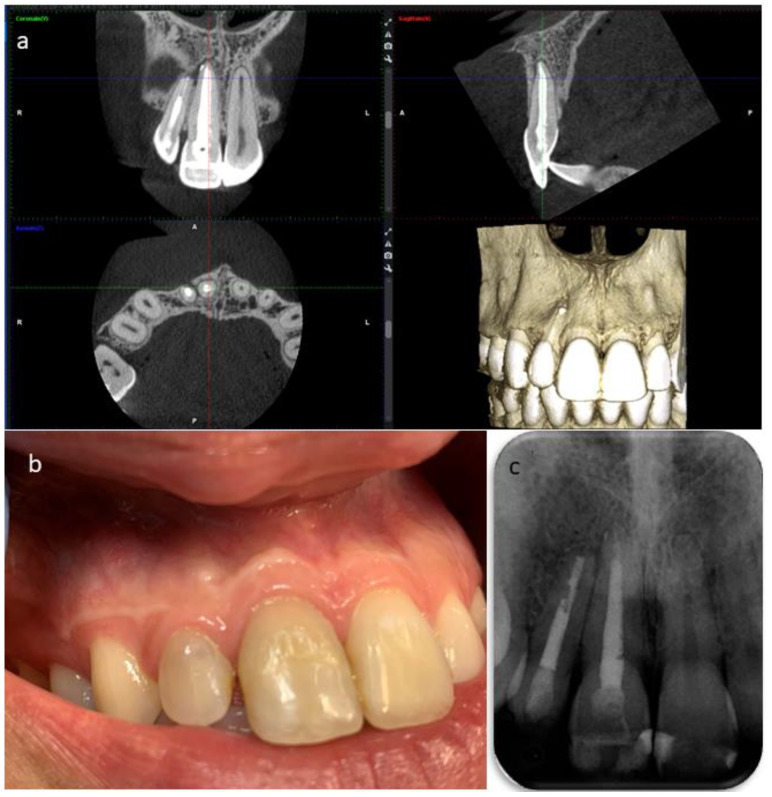
Follow-up at three years. (**a**) Post-operative CBCT performed at three years. (**b**) Clinical examination at three years revealing healthy soft tissues at the periapical area. (**c**) Periapical radiograph taken after three years postoperatively.

The secondary root canal treatment was performed in two visits. At the first appointment, the endodontic filling was gently removed, under microscope magnification, using K-files and a solvent (Endosolv E, Septodont, New Castle, DE, USA). Chemical disinfection with 5% sodium hypochlorite was used throughout the procedure, an apical gauging of 40 was determined with the apex locator and the canal was filled with calcium hydroxide paste, and the access cavity was sealed with a temporary cement. At the second appointment, the root canal was re-accessed, disinfected with the addition of a final rinse of 17% EDTA, and obturated with gutta-percha and zinc-oxide eugenol cement using the continuous wave technique, but the quality of apical instrumentation was deemed not satisfactory due to difficulties in completely removing the previous fill and reaching the full working length. The access cavity was restored with composite resin (Figure 4), and it was planned to wait three months before a surgical endodontic treatment. At the follow-up, the patient was still sensitive to chewing, a swelling was palpable in the alveolar mucosa between teeth 1.2 and 1.1, and tooth 1.1 still gave an uncertain response to thermal testing. In addition, the sinus tract was still visible (Figure 5). A second ultrasound examination showed that the sinus tract pathway was still present, and exhibiting a high degree of inflammation (Figure 5). The periapical surgery was then scheduled, and a preoperative cone-beam computed tomography (CBCT) was made to understand the size of the lesion and the involvement of the roots and alveolar bone. Scanning in the three planes showed large bone loss of the periapical areas of teeth 1.2 and 1.1 (Figure 5). Following the administration of local anesthesia, a sub-marginal flap was elevated, the root apex was located, and the periapical lesion removed carefully in order to preserve the tooth. In addition, the bone cavity was examined and no involvement of the apex of tooth 1.1 was diagnosed. The root-end was resected for approximately 3 mm, using a fissure bur, and a retrograde obturation was performed with a bioactive cement (Pro-Root MTA, Dentsply Tulsa Dental, Tulsa, OK, USA) (Figure 6). After fixation in 10% formalin, the surgical specimen was routinely processed for histopathologic assessment, revealing the presence of an apical abscess within granulomatous tissue, thus confirming the echographic diagnosis. At the one month recall, the patient reported the persistence of the active sinus tract and the ultrasound imaging confirmed the findings of the previous examination (Figure 6). Periodontal probing was repeated to exclude the presence of a vertical root fracture on tooth 1.2, and a cavity test was performed on tooth 1.1, which was then diagnosed with pulpal necrosis and treated endodontically. Upon opening of the access cavity, and during the chemo-mechanical disinfection of the endodontic space, there was a drainage of purulent material from the canal. The procedure was accomplished in a single visit. The canal was instrumented manually with continuous irrigation with 5% sodium and a final irrigation with 17% EDTA solution. After the confirmation of an apical diameter of 35 with gauging performed with the electronic apex locator, obturation was performed using the continuous wave technique of gutta-percha compaction and a zinc oxide-eugenol root canal sealer (Pulp Canal Sealer, Kerr, Romulus, MI, USA). After a week, it was possible to document the complete regression of clinical signs and symptoms in the involved teeth, as well as the disappearance of the stoma. A last ultrasound examination showed the disappearance of the bony tract and reduction of the Doppler inflammatory signal (Figure 7). The restoration of both teeth was performed with composite resin. The patient attended recall visits for three years, remaining asymptomatic and exhibiting radiographic healing of the lesions (Figure 8).

## 3. Discussion

The present report documents the sequelae of a sport related dental trauma where a multi-modular assessment involving ultrasound examination was necessary in order to correctly manage the case. Traumas of the oral-dental region have a considerable social impact, as these events represent 5% of all injuries for which patients require treatment. Among the multiple long-term complications of dental trauma are apical periodontitis, internal or external root resorption, and vertical root fractures, which can result in the loss of the tooth [16]. The possibility of implementing clinical diagnostic tests and traditional periapical radiographs alongside advanced imaging systems, such as CBCT and ultrasounds, is of paramount importance to manage trauma cases, especially when multiple teeth are involved and there is the risk of overtreatment. In general, sensitivity thermal tests are highly reliable; cold and heat testing exhibit a comparable sensitivity (0.83 and 0.86, respectively). However, heat tests show a drop in specificity (0.43), whereas in cold tests this measure appeared even higher (0.93). Considering these results, the overall accuracy of cold and heat testing has been calculated to be 0.86 and 0.71, respectively [17,18]. Furthermore, cold and heat tests are generally less reliable in traumatic dental injuries, where their sensitivity is slightly higher (0.84 and 0.87), but the drop in specificity is important (0.76 and 0.61, respectively). Based on these data, the overall accuracy in dental traumas is 0.78 for cold and 0.68 for heat tests [19]. This is attributable to the vascular and neural damage to the involved teeth, which are difficult to evaluate in close proximity to the trauma. It is interesting to consider that approximately 80% of teeth that exhibit an initial positive response to pulp sensitivity tests are vital at the final recall. In addition, since neural regeneration in traumatized teeth is slower than vascular regeneration, or even absent, the teeth may remain vital even though they do not respond to tests [19]. The possibility of induced pulpal necrosis after surgery was considered; however, it should be taken into consideration that the symptomatology remained stable from the beginning until the completion of the root canal treatment on tooth 1.1 in this case; for this reason, this option should be discarded.

In the management of the present case, the contradictory responses of tooth 1.1 to sensitivity, in conjunction with the lack of healing of AP after both orthograde and surgical endodontic treatment, led the clinical team to consider whether to treat tooth 1.1 or extract tooth 1.2. However, it is well known that edentulism, even when affecting a single tooth (whether congenital or the result of a traumatic injury) is an extreme type of damage and should require early intervention, either prosthetic, orthodontic or conservative, in adolescents and young adults, to avoid long term complications [20,21]. Given the young age of the patient, in the case of extraction, the therapeutic alternatives considered were either a fixed partial denture or a Maryland bridge as a viable alternative to an implant-supported crown, to await higher bone stability and preserve this option for the future. The objectivation of a sinus tract in a trauma case is of great interest, because the presence of a pre-operative sinus tract has been reported to decrease the odds of healing after endodontic treatment by 48%, and to increase the probability of tooth loss by 120% [6,22]. The obstacle in the healing of sinus tract-associated lesions was explained recently in a histo-bacteriologic study on 24 biopsy specimens, where the authors highlighted the complex infectious patterns of those cases. It was reported that 83% of the samples examined had a complex infectious pattern that comprised the canal system, the periapical lesion, and the extra-radicular portion of the root; this was always derived from the intra-radicular biofilm [23]. The traditional diagnostic approach to disclose the origin and pathway of sinus tracts is based on the insertion of an orthodontic wire or a gutta-percha cone through the opening of the tract, followed by a periapical radiograph [24,25]. This procedure is invasive, it may be tricky, and it can create discomfort to the patient; moreover, it does not provide information on the inflammatory state of the area. It also does not provide a direct image of the route of pus drainage, and the lesion can be distorted according to the level of flexibility of the gutta-percha cone. Therefore, ultrasound examination can be used to directly visualize sinus tracts of endodontic origin, and to trace their pathways. In addition, as demonstrated by previous studies, ultrasound examination with CPD was useful for the monitoring of the periapical lesion and the sinus tract one week from the completion of the root canal treatment. Indeed, it was possible to evaluate not only the disappearance of the fistulous tract, but also the reduction of the vascular signal, which is related to resolution of the inflammatory process [12,13].

## 4. Conclusions

This work showed the importance of a multi-modular assessment in the diagnosis, treatment, and follow-up of the sequelae of a sport-related dental trauma. The traditional periapical radiographs were used, as they still represent the gold standard to disclose the presence of the lesions in the bone and of previous endodontic treatments. They are also useful during treatment and for follow-up. CBCT was of utmost importance to observe the size and spatial relationship of the lesion to the surrounding anatomical structures, to plan the endodontic and surgical interventions, and to accomplish a predictable long-term follow-up. The present report further demonstrated the potential use of ultrasound imaging as a diagnostic aid in the maxillofacial district. Indeed, ultrasound examination was of particular interest in non-invasively visualizing and tracing the sinus tract, and revealing the content of the lesion. In addition, this latter technique was demonstrated to be reliable not only in monitoring the periapical lesion, but also in predicting the long-term outcome of endodontic treatment [12,13] based on the early echographic response of the lesion at one week after treatment.

## Data Availability

The data used during the current study are available from the corresponding author on reasonable request.
